# A Bacteriological Comparison of the Hemolymph from Healthy and Moribund Unionid Mussel Populations in the Upper Midwestern U.S.A. Prompts the Development of Diagnostic Assays to Detect *Yokenella regensburgei*

**DOI:** 10.3390/microorganisms11041068

**Published:** 2023-04-19

**Authors:** Eric M. Leis, Sara Dziki, Isaac Standish, Diane Waller, Jordan Richard, Jesse Weinzinger, Cleyo Harris, Susan Knowles, Tony Goldberg

**Affiliations:** 1 La Crosse Fish Health Center—Midwest Fisheries Center, U.S. Fish and Wildlife Service, Onalaska, WI 54650, USA; sara_dziki@fws.gov (S.D.); sirisaac_standish@fws.gov (I.S.); 2 U.S. Geological Survey, Upper Midwest Environmental Sciences Center, La Crosse, WI 54603, USA; dwaller@usgs.gov; 3 Department of Pathobiological Sciences and Freshwater & Marine Sciences Program, University of Wisconsin-Madison, Madison, WI 53711, USA; jcrichard2@wisc.edu; 4 Southwestern Virginia Field Office, U.S. Fish and Wildlife Service, Abingdon, VA 24210, USA; 5 Wisconsin Department of Natural Resources, Madison, WI 53703, USA; jesse.weinzinger@wisconsin.gov; 6 Michigan Department of Natural Resources, Waterford, MI 48327, USA; harrisc9@michigan.gov; 7 U.S. Geological Survey, National Wildlife Health Center, Madison, WI 53711, USA; sknowles@usgs.gov

**Keywords:** *Yokenella regensburgei*, Embarrass River (WI), Huron River (MI), St. Croix River (WI), mussel mortality events, molecular assays

## Abstract

Recent bacteriological investigations of freshwater mussel mortality events in the southeastern United States have identified a variety of bacteria and differences in bacterial communities between sick and healthy mussels. In particular, *Yokenella regensburgei* and *Aeromonas* spp. have been shown to be associated with moribund mussels, although it remains unclear whether these bacteria are causes or consequences of disease. To further understand the role of bacteria in mussel epizootics, we investigated mortality events that occurred in the upper Midwest in the Embarrass River (Wisconsin) and the Huron River (Michigan). For comparison, we also studied mussels from an unaffected population in the St. Croix River (Wisconsin). Diverse bacterial genera were identified from these sites, including *Y. regensburgei* from moribund mussels in the Embarrass River (Wisconsin). This bacterium has also been consistently isolated during ongoing mortality events in the Clinch River (Virginia). Subsequently, we developed and validated molecular assays for the detection of *Yokenella* to use in future investigations of mussel mortality events and to identify environmental reservoirs of this bacterium.

## 1. Introduction

Freshwater mussels (Unionidae) represent an important component of healthy freshwater ecosystems as they provide a number of services that improve conditions in the aquatic environment [1]. These animals are also among the most imperiled in the world with more than 70% of the species considered threatened, endangered, or of special concern due to a variety of factors, including pollution, water quality degradation, habitat alteration, and invasive species, among others [2,3]. Recently, enigmatic declines of freshwater mussel populations have been observed where no clear cause can be attributed, and other aquatic fauna appear unaffected [4]. While diseases are often considered to be involved, until recently there have been few microorganisms connected with mussel mortality events [4,5,6,7,8,9,10,11]. Studying bacterial communities associated with freshwater mussels is complex given that mussels feed on bacteria in an everchanging aquatic environment, and diverse bacteria have been associated with healthy mussels [5,6,12,13]. Furthermore, bacterial communities likely shift rapidly once mussels become moribund and die [5,8]. Due to these factors, as well as a general dearth of information about the biology of these ecologically important animals, little is known about the bacterial pathogens of freshwater mussels [5]. Additional studies producing data regarding baseline bacterial communities could ultimately be useful in determining the bacterial pathogens of unionids [6].

The most recent culture-based bacteriological evaluations of mortality events involving pheasantshell mussels (*Ortmanniana pectorosa*; syn: *Actinonaias pectorosa*) in the Clinch River (Virginia/Tennessee, USA) identified two bacterial genera, *Yokenella* and *Aeromonas*, that were associated with moribund mussels [6,10]. Of these, the most intriguing was *Yokenella regensburgei* [6,8,10], due to its relatively high prevalence, strong statistical association with moribund mussels, and a nearly exclusive association with active mortality events. Richard et al. [8] supported these findings through a metagenomic microbiome analysis and showed that mussels identified as healthy had a lesser general bacterial load than did moribund mussels, an observation that was also made through a culture-based evaluation by Starliper et al. [14]. While *Y. regensbergei* has been associated with human infections in immunocompromised individuals (see discussion in [15]), the only associations of the bacterium with morbidity or mortality in aquatic animals have been with pheasantshell mussels from the Clinch River [6,8,10] and a recent report of septicemia in alligators (*Alligator mississippiensis*) [15]. To continue to assess the bacterial communities associated with freshwater mussels, as well as to determine if *Y. regensburgei* could be detected in mortality events outside of the southeastern United States, we cultured bacteria from mussel hemolymph during two midwestern mortality events located in the Embarrass River (Wisconsin) and Huron River (Michigan) and compared them with samples collected from an apparently healthy control site at the St. Croix River (Wisconsin). Due to the apparent significance of *Y. regensburgei*, molecular assays were developed and validated to detect this bacterium. These assays have future applications examining mussel mortality events, as well as searching for potential sources and environmental reservoirs of this bacterium. 

## 2. Materials and Methods

### 2.1. Study Sites

**Figure 1:**
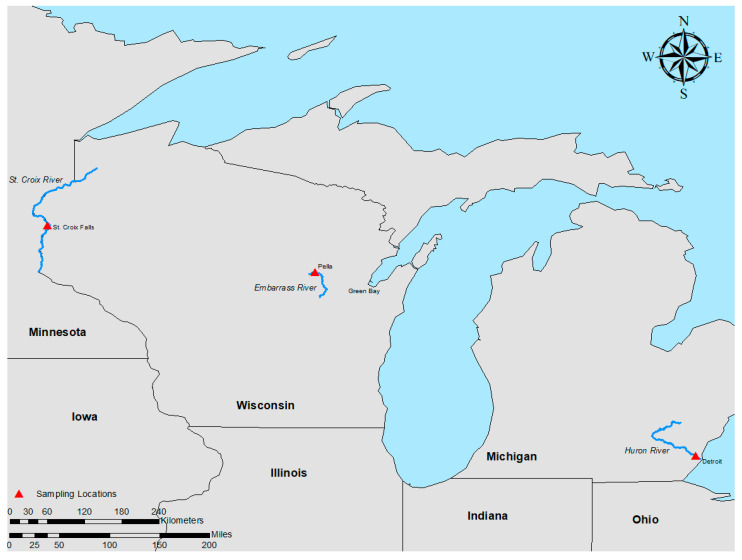
Map of sampling locations, which includes the Huron River (MI), Embarrass River (WI), and St. Croix River (WI).

Freshwater mussels were collected from rivers in the midwestern United States (Figure 1). Healthy mussels were characterized as displaying typical behavior, including being partially buried in the sediment and exhibiting siphoning behavior, as well as quick responses to handling. Mussels were considered moribund if they were laying on their side at the surface of the riverbed and were slow to respond to handling. 

#### 2.1.1. Embarrass River (Wisconsin; 44°44′23.44″ N; 88°47′54.94″ W) 

The Embarrass River (Shawano County, WI) is a medium-sized river with a mean width of 51 m (Figure 1). Current velocities average 0.46 m/s, with a maximum of up to 1.2 m/s, and water depths averaging 44 cm. The sediment is dominated by heterogeneous gravel, with sand and cobble present throughout. The unionid community consists of 24 species, including *Epioblasma triquetra*, a U.S. federally endangered species. Total live mussel density is estimated at 12.6 (±1.11) individuals/m^2^.

In June of 2018, Wisconsin Department of Natural Resources staff observed significant mussel mortality below the dam at Pella (44°44′23.44″ N; 88°47′54.94″ W) during scheduled monitoring. Mussel mortality was not limited to one or a few species and was evenly distributed throughout the river’s varying depths. Mussel mortality ranged 3 km downstream from the first reported records. A follow-up site visit occurred in October of 2018, and moribund mussels were observed. The water temperature was 9 °C. Examination of historical records revealed that a similar mortality event was discovered in 2014 during a four-person hour survey at the same site [16]. Similar mortality was not observed in stream segments above the Pella Dam, indicating that the negative effects may have been related to dam drawdown events. 

During the mortality event sampling that occurred in October 2018, hemolymph samples were collected in the field (as described below) from the following mussels: 8 mucket (*Actinonaias ligamentina*; syn: *Ortmanniana ligamentina*; 4 moribund, 4 healthy), 4 deertoe (*Truncilla truncata*; 4 moribund), 4 plain pocketbook (*Lampsilis cardium*; 2 moribund, 2 healthy), 4 pink heelsplitters (*Potamilus alatus;* 2 moribund, 2 healthy), 2 fat mucket (*Lampsilis siliquoidea*; 1 moribund, 1 healthy), and 2 fragile papershell (*Leptodea fragilis*; 1 moribund, 1 healthy). 

#### 2.1.2. Huron River Sampling (Michigan; 42°5′19.79″ N; 83°16′57.98″ W) 

On 9 September 2019, the Michigan DNR received notice of a possible mussel mortality event in the Huron River (Oakland County, MI) (Figure 1). A field investigation the next day found three muckets that were alive and 70 that appeared recently deceased. On 10 September 2019, an additional site located 1 km upstream was briefly investigated and no freshly dead mussels were found, but only one live mussel (mucket) was observed, along with many weathered shells that could not be identified. On 11 September 2019, we returned to the initial mortality event location. Deep water made observations difficult, even with an aquascope, so a snorkeling effort was undertaken. Fifteen healthy and four moribund muckets were collected from the Huron River (size range 76 to 144 mm), wrapped in wet towels, and sent overnight to the La Crosse Fish Health Center for sampling, as described below. The search area of the stream covered about 25.9 linear m × 25.9 m wide. Stream depth ranged from 0.2 to 0.9 m in the search area. During both efforts, Cyprinidae and Gobiidae fish species, as well as other unionid species, were observed alive and in normal condition. Water temperature was 23.6 °C. 

One year later (23 September 2020), the site was revisited, and 21 apparently healthy muckets (size range 67 to 136 mm) were collected. No moribund mussels were found. This effort covered a similar area as in 2019 and extended upstream an additional 20 m. Stream depth ranged from 0.1 to 0.8 m. Water temperature was 18.3 °C.

#### 2.1.3. St. Croix River Sampling (Wisconsin; 45°23′56.03″ N; 92°38′59.53″ W)

The St. Croix River, located in Polk County along the western edge of Wisconsin, was chosen as a reference site because it is known to have a stable and diverse mussel population (Figure 1). Twelve apparently healthy muckets (size range 65 to 103 mm shell length) were collected by hand on 21 August 2018, and hemolymph was collected in the field (as described below). Water temperatures ranged from 22 to 24 °C. The population appeared healthy, and no signs of disease were observed at the time of sampling. 

### 2.2. Hemolymph Sample Collection

For all sites, samples were collected as described in Leis et al. [6]. Briefly, hemolymph was drawn from the anterior adductor muscle of each mussel with a sterile needle (31 ga) and syringe (1 mL). Two drops (~100 µL) of hemolymph were immediately plated onto Tryptic Soy Agar (TSA), incubated at 21 °C for 7 to 14 days, and phenotypically unique colonies were picked with a disposable 1 ul loop and placed into a sterile microcentrifuge tube. The isolates were then extracted with Prepman Ultra Sample Preparation Reagent (Thermo Fisher Scientific, Waltham, MA, USA) and underwent molecular analysis via 16S rRNA PCR with primers used in Leis et al. [6]. The products of successful reactions were Sanger sequenced (Eton Biosciences; Union, NJ, USA), and the resulting sequences were assembled in Geneious (www.geneious.com) and identified through BLAST searches in Genbank [17]. 

### 2.3. Development of Yokenella-Specific Diagnostic Assays

#### 2.3.1. qPCR Development and Evaluation

A whole genome sequence for *Y. regensburgei* (CP050811.1) in Genbank was used to design primers (Forward: 5′-ATGTCACATCTCGCAGAGCTGGT-3′; Reverse: 5′-ACTGTTTGAGGAAACGCAGATCG-3′) targeting the phenylalanyl-tRNA synthase alpha subunit (*pheS*) gene for this bacterium. Isolates from previous studies in the Clinch River identified as *Y. regensburgei* (samples 77A, 28A, and 28gA from 25 October 2018; see Supplementary Material in [10]) were extracted using the PrepMan Ultra Sample Preparation Reagent (Thermo Fisher Scientific) according to the manufacturer’s protocol and underwent PCR amplification of the *pheS* gene using the new primers. The sequences obtained for these three *Yokenella* isolates (Genbank Accession OQ831664, OQ831665, and OQ831666) were used to design a qPCR assay. The *Yokenella* qPCR was designed, optimized, and performed as previously described [18]. Briefly, the 100 bp amplicon was targeted using the primers (*Yokenella*-qPCR Forward) 5′-CTGAATATTCCGGGACACCAT-3′, (*Yokenella*-qPCR Reverse) 5′-GGATCTGAACGCCAGAAGTC-3′, and probe (*Yokenella*-qPCR Probe) 5′-FAM/ACCACGACACCTTCTGGTTTGATG/IABkFQ -3′. The probe contained a ZEN internal quencher and a 3′ Iowa Black^®^ fluorescent quencher (Integrated DNA Technologies, Inc., Coralville, IA, USA). All reactions (25 μL) were conducted using 200 nM of both primers, 400 nM of probe, and 12.5 μL of PrimeTime^®^ Gene Expression MasterMix (Integrated DNA Technologies, Inc., Hercules, CA, USA) in a CFX96 Touch™ (Bio-Rad Laboratories, Hercules, CA, USA) with the following cycling conditions: 2 min at 50 °C and 10 min at 95 °C, followed by 40 cycles of 95 °C for 15 s and 60 °C for 1 min.

**Figure 2:**
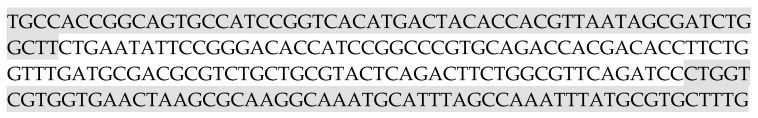
*Yokenella pheS* gBlock^®^ sequence. The gBlock^®^ contains a 100 bp portion of the *Yokenella* phenylalanyl-tRNA synthase alpha subunit (*pheS*) gene flanked by nonspecific protective bases highlighted in grey.

A synthetic gene block (gBlock^®^; Figure 2) was used for optimization and quantification purposes. The gBlock^®^ was rehydrated, quantified, and diluted as described previously by Standish et al. [19], resulting in a standard curve of 10-fold dilutions ranging from 10^7^ to 1 copy reaction^−1^. Optimal primer and probe concentrations, assay variability, the limit of detection (LOD), and limit of quantification (LOQ) were identified as previously described [19]. 

Efficiency and repeatability were evaluated using eight replicate reactions of each concentration of the gBlock^®^ standard curve (intra-assay variation), then compared to eight replicates of the standard curve on a subsequent plate to determine inter-assay variation by evaluating the mean, standard deviation, and coefficient of variation (CV). The Baseline Subtracted Curve Fit Mode in the Bio-Rad CFX v3.0 (Bio-Rad Laboratories) software was used to calculate the reaction efficiency, slope, and R^2^ values across triplicate plates containing the standard curve. 

The synthetic gBlock^®^ was also used to evaluate the qPCR sensitivity. The LOD—defined as the lowest target concentration where amplification was observed in >95% of replicate reactions—was determined by running 20 reactions containing 100, 10, and 1 copy reaction^−1^. The LOQ was evaluated by running eight replicates of the gBlock^®^ (10^7^ to 1 copy reaction^−1^). Standard values were assigned to 4 replicates, and the remaining 4 were used to calculate the starting quantity (SQ) mean, SQ standard deviation, and CV. The LOQ was defined as the lowest concentration with an SQ CV < 25% [20]. 

Assay specificity was evaluated against isolates of fish bacterial pathogens as well as other common and/or related bacterial species. Isolates were either obtained from ATCC (Manassas, VA, USA) or from the La Crosse Fish Health Center archive (Midwest Fisheries Center, La Crosse, WI, USA). Isolates of *Aeromonas salmonicida, Yersinia ruckeri* (ATCC^®^ 29473), *Edwardsiella ictaluri*, *Chryseobacterium aahli*, *Flavobacterium columnare*, *Vagococcus salmoninarum* (ATCC^®^51200), and *Carnobacterium maltaromaticum* (ATCC^®^ 27865) were examined. Specificity was also evaluated against additional representative species: *Enterobacter aerogenes*, *Escherichia coli, Shigella flexneri*, *Staphylococcus epidermidis*, *Lactobacillus paracasei, L. rhamnosus*, *Proteus vulgaris,* and *Vagococcus lutrae* (ATCC^®^ 700839). Additionally, assay specificity was tested against closely related bacteria and was examined through analysis of DNA from the following species: *Klebsiella pneumonia*, *Shewanella oneidensis*, *Morganella morganii*, and *Salmonella enterica*, as well as isolates of *Kluyvera intermedia* (ATCC^®^ 33423), *Serratia marcescens* (ATCC^®^ 14756), *Enterobacter cloacae* (ATCC^®^ 13047), *Citrobacter freundii* (ATCC^®^ 8090), *Hafnia alvei* (ATCC^®^ 29926), and *Buttiauxella izardii* (ATCC^®^ 51606). Finally, the three individual *Yokenella* sp. isolates recovered from mussel epizootics in the Clinch River, VA, USA (see [6]) and used in assay development were used to validate specificity. 

#### 2.3.2. End-Point Conventional PCR (cPCR) Development and Evaluation

A second end-point cPCR was designed as a confirmatory assay. The *Yokenella* end-point PCR also targeted the *pheS* gene, amplifying a 597 bp portion of the gene using the forward primer 5′-GATCGACGTTTCTCTTCCTGGC-3′ and reverse primer 5′-ATAAACTTCCGGGTCGATGCCG-3′. Reactions (50 μL) contained 200 nM of each primer and Platinum PCR SuperMix (Thermo Fisher Scientific), and 3 µL of template DNA with the following cycling conditions: 95 °C for 5 min; 35 cycles of 95 °C for 30 s, 53 °C for 30 s, and 72 °C for 60 s; and a final extension at 72 °C for 7 min. Reactions were visualized using the E-Gel Electrophoresis System (Thermo Fisher Scientific) and compared to bands of the TrackIt™ 100 bp ladder (Thermo Fisher Scientific).

End-point cPCR specificity was evaluated against the individual *Yokenella* sp. isolates and the following closely related Enterobacteria species: *Klebsiella pneumonia*, *Shewanella oneidensis*, *Morganella morganii*, *Salmonella enterica*, and isolates of *Yersinia ruckeri* (ATCC^®^ 29473), *Kluyvera intermedia* (ATCC^®^ 33423), *Serratia marcescens* (ATCC^®^ 14756), *Enterobacter cloacae* (ATCC^®^ 13047), *Citrobacter freundii* (ATCC^®^ 8090), *Hafnia alvei* (ATCC^®^ 29926), and *Buttiauxella izardii* (ATCC^®^ 51606). 

### 2.4. Molecular Evaluation of Bacterial Isolates

All bacteria samples collected from the Embarrass River were also analyzed to further validate the qPCR and cPCR assays against cultures of various genera. Any samples positive by qPCR underwent evaluation by the end-point cPCR. Any positives by cPCR were Sanger sequenced using the primers used during amplification (Eton Biosciences; Union, NJ and Eurofins Genomics; Lousiville, KY, USA). Sequences were assembled and identified through BLAST searches in Genbank [17].

## 3. Results

### 3.1. Survey Results and Bacterial Isolation

A total of 76 mussels were sampled from the three sites, and 30 bacterial genera were identified from the mussels surveyed in the upper Midwest (Table 1). Overall, 21/30 of the genera were found in a single sampling event, of which 16/21 occurred as single-incident isolates and 6/30 genera occurred in two sampling events. The remaining three genera—*Aeromonas*, *Bacillus*, and *Pseudomonas*—were identified from all four sampling events. In total, bacteria were identified from 61% (46 of 76) of the mussels sampled. From the St. Croix River, bacteria were isolated and identified from 100% (12 of 12) of the apparently healthy muckets (Table 1). Fourteen genera were identified, and *Exiguobacterium* spp. (53%; 8 of 12) and *Bacillus* (33%; 5 of 12) had the highest prevalence. During the mortality event in the Huron River (2019), bacteria were cultured from 42% (8 of 19) of muckets yielding nine genera, and the bacterium identified with the highest prevalence was *Aeromonas* spp. (21%; 4 of 19). The following year in the Huron River, when active mortality was not occurring, we found 12 genera, most of which were single-incident isolates (Table 1). During the mortality event in the Embarrass River, WI, bacteria were isolated from 83% (20 of 24) mussels sampled, yielding 10 genera. We identified *Y. regensburgei* from four mussel species including 13% (1 of 8) of muckets, 25% (1 of 4) of plain pocketbooks, 50% (2 of 4) of pink heelsplitters, and 50% (2 of 4) of deertoe. Additionally, *Aeromonas* was identified from 54% (13 of 24), and *Pseudomonas* was identified from 25% (6 of 24) of the Embarrass River mussels. 

### 3.2. Assay Development

#### 3.2.1. qPCR Assay

The *Yokenella* qPCR was efficient, repeatable, and sensitive (Table 2 and Table 3). Across duplicate plates containing the gBlock^®^ standard curve, the assay yielded low CV values (<2%) for both intra- and inter-assay variation (Table 2). Across triplicate plates, linear regression of Cq values versus log_10_ gBlock^®^ demonstrated an average R^2^ value of 0.997 (SD = 0.003), a slope of −3.269 (SD = 0.062), and an average efficiency of 101.6% (SD = 3.6).

The LOD and LOQ were determined as 10 copies. Amplification was observed in 100% of the twenty replicate reactions containing one hundred and ten copies and in seven of twenty reactions containing one copy. Additionally, the SQ CV values were 18.68% for ten copies, but because of a lack of amplification, they could not be determined for one copy (Table 2). The qPCR assay was specific; however, nonexponential amplification was observed with two closely related bacteria species: *Klebsiella pneumonia* and *Morganella morganii*. 

#### 3.2.2. End-Point cPCR Assay

The end-point cPCR exhibited a robust specificity, with a strong band at 596 bps for the *Yokenella* isolates examined during the initial assay development. Minor nonspecific banding was observed with closely related species: 150 bps for Serratia marcescens; 250 bps for *Morganella morganii*; multiple faint bands at 250, 1000, and 1100 bps for both *Salmonella enterica* and *Shewanella oneidensis*. A single band at ~600 bps was used for positive discrimination. 

#### 3.2.3. Molecular Evaluation of Bacterial Isolates

The qPCR and cPCR assays targeting the *pheS* gene confirmed the presence of *Y. regensburgei* in samples that were identified as such with 16S rRNA sequencing from the Embarrass River (see Table S1). Four additional isolates were positive by both *Yokenella*-specific assays, despite being identified as other bacterial genera by the initial 16S rRNA sequencing (Table 1). Sequencing of the cPCR product revealed that this was likely due to a combination of assay sensitivity and a low level of contamination that occurred while picking colonies. The positive cPCR bands were sequenced, and the resulting contigs were all >95.9% similar to the *pheS* gene of *Y. regensburgei* (Genbank Accession: CP050811.1) and not to other bacterial genera (see Table S1).

## 4. Discussion

The healthy reference mussels from the St. Croix River yielded the highest diversity of bacterial genera and had a significantly higher average number of genera per mussel than those from the Huron River. *Exiguobacterium* spp. and *Bacillus* spp. were identified with high prevalence from these healthy reference mussels in the St. Croix River, a finding consistent with a report from the upper Mississippi River [6], which is connected to the St. Croix River. Determining whether these potentially superficial connections are legitimate or simply an artifact of low sample numbers across limited seasons could be the focus of future studies. The identification of bacteria consistently associated with healthy individuals could have value in evaluating the health status of mussel populations. It is also possible that some isolates may represent beneficial bacteria, although any positive associations became masked by their presence in moribund mussels. For example, if a pathogenic or opportunistic bacterial infection does not fully displace others in the hemolymph, the microbes that positively influence mussel health may not be easily discerned. In vivo trials could be used to evaluate whether these isolates might be useful probiotics with applications related to rearing robust individuals in propagation facilities.

Our investigations of mortality events in the Huron River and Embarrass River identified *Aeromonas* with a high prevalence in dying mussels, and a year later, follow-up sampling of the Huron River again identified *Aeromonas* but with a lower prevalence. This finding is very similar to a report from a pheasantshell mortality event in the Clinch River where *Aeromonas* was more prevalent during active epizootics [6,8,10]. Interestingly, this bacterial genus was not identified in St. Croix River mussels and was found at a relatively low overall prevalence from apparently healthy mussels in the upper Mississippi River [6]. While *Aeromonas* typically represents environmentally ubiquitous, opportunistic invaders, this taxonomic group also contains some species considered primary pathogens of aquatic organisms [21,22]. Our data to date may suggest that *Aeromonas* opportunistically invades mussel hemolymph during times of stress and/or when immunocompromised. Future work could further evaluate whether any *Aeromonas* strains are primary or secondary pathogens of unionids.

The identification of *Y. regensburgei* from the Embarrass River represents an important finding. This bacterium has now been consistently identified in mortality events involving pheasantshells in the Clinch River (VA/TN; [6,8,10]) and from a mortality event of Ebonyshells (*Fusconaia ebena*) in the Tennessee River ([14]; also see discussion in [10]). This bacterium has only been identified from sites known to have mortality events, and only a single isolate has been identified outside of an active mussel mortality event, although its isolation occurred a short time before mortalities began [6,10]. This appears to be the first isolation of this bacterium from mussels outside of the southeastern United States. Previous studies of recurring mortality events in the Clinch River (Tennessee/Virginia, USA) have found patterns of high co-occurrence of *Yokenella* and *Aeromonas* in moribund mussels [6,8,10]. In the Embarrass River, 62.5% of samples had *Yokenella*, *Aeromonas*, or both. From these samples, the two instances of *Yokenella* present in apparently healthy mussels occurred without any other bacteria present. In the four instances of *Yokenella* occurring in moribund mussels, it co-occurred with *Aeromonas* and/or *Pseudomonas* in 3/4 cases. The association of *Yokenella* with a mortality event in the midwestern U.S. leads to questions regarding whether there is a common factor that connects all the mortality events where *Y. regensburgei* has been isolated, as it is unclear whether this microbe is pathogenic or possibly a bioindicator of contamination [6,10]. In the Embarrass River, the only observation that seemed to support the potential for a chemical stressor being present was related to a buildup of some sort of foam in the bends of the river. While foam is typically associated with natural processes, it is still worth noting as it can also be related to anthropogenic inputs [23]. Whatever the cause of the mortality event in the Embarrass River, future work evaluating the association of *Y. regensburgei* with mortality events could provide insight.

The qPCR assay presented herein is both sensitive and specific for the detection of *Y. regensburgei*, especially when paired with cPCR confirmation and sequencing. The assay confirmed the identity of *Y. regensburgei* from all the samples identified as this bacterium through the initial 16S rRNA sequencing. In addition, four other isolates were also positive by the molecular assays developed herein to detect *Yokenella*. Sequencing the products of the cPCR assay indicated that these positives were undoubtably attributed to low levels of contamination, as they all sequenced as the *pheS* gene of *Yokenella*. It seems likely that imperceptible *Yokenella* cells were adjacent to or obscured by targeted isolates when colonies were picked from the plates, and the assay amplified this small amount of contamination. We tested the assay against cultures obtained from the Embarrass River as well as pure isolates of related species to fully test the specificity and functionality of these assays, putting them through a rigorous challenge. When used as described here, these assays are ready to be applied to evaluate mussel mortality events (mussel tissues, hemolymph, pore water, sediments, etc.) in greater detail as well as to search for sources and/or reservoirs of this bacterium in the environment. In addition to the field-based applications, using these assays to evaluate laboratory infections could have real merit in determining whether the consistent identification of this bacterium during some mussel mortality events is incidental or if a more pathogenic association is present.

## 5. Conclusions

*Yokenella regensburgei* was identified during a mussel mortality event in the Embarrass River, Wisconsin. This is significant given the consistent association between the presence of this microbe and pheasantshell mortality events in the southeastern United States [6,8,10]. The reasons for this association remain unknown; however, the molecular assays developed herein may be useful in searching for any verifiable relationship between moribund mussels and this bacterium.

## Figures and Tables

**Table 1:** Bacterial genera isolated from the hemolymph of mussel species from rivers in the Midwestern United States.

Sampling Date	Location	Mussel Species	Number Sampled	Genus Identification	Prevalence
**21 August 2018**	**St. Croix River**	**Mucket**	12	*Acinetobacter*	2/12
		*Actinonaias ligamentina*		*Aeromonas*	1/12
				*Arthrobacter*	2/12
				*Bacillus*	5/12
				*Brevundimonas*	1/12
				*Ensifer*	1/12
				*Enterobacter*	1/12
				*Exiguobacterium*	7/12
				*Microbacterium*	1/12
				*Paenibacillus*	1/12
				*Pseudarthrobacter*	1/12
				*Pseudomonas*	2/12
				*Rhodococcus*	1/12
				*Terracoccus*	1/12
**5 October 2018**	**Embarrass River**	**Mucket**	8	*Aeromonas*	3/8
		*Actinonaias ligamentina*		*Chitinibacter*	1/8
				*Flavobacterium*	1/8
				*Pseudomonas*	1/8
				*Yokenella*	1/8
		**Plain Pocketbook**	4	*Aeromonas*	2/4
		*Lampsilis cardium*		*Yokenella*	1/4
		**Fragile Papershell**	3	*Aeromonas*	1/3
		*Leptodea fragilis*		*Bacillus*	1/3
				*Flavobacterium*	1/3
				*Pseudomonas*	1/3
		**Fat Mucket**	2	*Aeromonas*	2/2
		*Lampsilis siliquoidea*		*Bacillus*	1/2
				*Chryseobacterium*	1/2
				*Pseudomonas*	1/2
				*Serratia*	1/2
		**Pink Heelsplitter**	4	*Aeromonas*	2/4
		*Potamilus alatus*		*Yokenella*	2/4
		**Deertoe**	7	*Aeromonas*	3/7
		*Truncilla truncata*		*Bacillus*	2/7
				*Curtobacterium*	1/7
				*Pseudomonas*	3/7
				*Sphingobacterium*	2/7
				*Yokenella*	2/7
**10 September 2019**	**Huron River**	**Mucket**	20	*Aeromonas*	4/20
		*Actinonaias ligamentina*		*Bacillus*	1/20
				*Erwinia*	1/20
				*Microbacterium*	1/20
				*Micrococcus*	1/20
				*Pseudomonas*	1/20
				*Rhodococcus*	1/20
				*Staphylococcus*	1/20
				*Vogesella*	2/20
**23 September 2020**	**Huron River**	**Mucket**	21	*Acidovorax*	1/21
		*Actinonaias ligamentina*		*Acinetobacter*	2/21
				*Aeromonas*	1/21
				*Bacillus*	1/21
				*Chromobacterium*	1/21
				*Chryseobacterium*	1/21
				*Delftia*	1/21
				*Flavobacterium*	1/21
				*Novosphingobium*	1/21
				*Pseudomonas*	1/21
				*Rheinheimera*	1/21
				*Staphylococcus*	1/21

**Table 2:** Repeatability of *Yokenella* qPCR assay targeting *pheS* gene. Table shows the mean, standard deviation (*SD*) and coefficient of variation (CV) of the cycle threshold (Cq) values. Amplification of serially diluted gBlock^®^, ranging from 10^7^ copies to 1 gene copy reaction^−1^. To determine intra-assay variation, eight replicates were conducted on a single plate. Eight replicates were conducted on a subsequent plate for determination of the inter-assay variation.

gBlock^®^ Copies	Cq Mean	Cq SD	Cq CV(%)
**Intra-assay**			
10,000,000	16.94	0.12	0.69
1,000,000	20.30	0.09	0.43
100,000	23.20	0.10	0.43
10,000	26.74	0.12	0.45
1000	29.68	0.09	0.29
100	33.30	0.13	0.40
10	36.60	0.31	0.84
1	39.24	0.38	0.96
**Inter-assay**			
10,000,000	17.15	0.25	1.46
1,000,000	20.34	0.19	0.91
100,000	23.02	0.29	1.25
10,000	26.54	0.44	1.65
1000	29.41	0.36	1.22
100	33.47	0.33	1.00
10	36.49	0.34	0.93
1	39.36	0.35	0.89

**Table 3:** *Yokenella* qPCR limit of quantification (LOQ). Assay LOQ was evaluated by running eight replicates of the *pheS* gene gBlock^®^ (10^7^ to 1 copy reaction^−1^). Standard values were assigned to 4 replicates, and the remaining replicates were used to calculate the starting quantity (SQ) mean, SQ standard deviation (SD) and SQ coefficient of variation (CV). There was insufficient amplification to perform calculations for 1 copy.

gBlock^®^ Copies	SQ Mean	SQ SD	SQ CV(%)
10,000,000	11,070,000.00	594,474.56	5.37
1,000,000	988,175.00	53,841.36	5.45
100,000	123,175.00	11,160.73	9.06
10,000	10,369.25	343.27	3.31
1000	1172.50	51.65	4.40
100	91.14	7.07	7.76
10	8.31	1.55	18.68
1	1.50	NA	NA

## Data Availability

Data is contained within the article and Supplementary Material.

## Supplementary Materials

The following supporting information can be downloaded at: https://www.mdpi.com/article/10.3390/microorganisms11041068/s1, Table S1: Identification data for each isolate according to study site as well as the results of BLAST searches for the sequence produced from each sample that was positive by the cPCR developed for the detection of *Yokenella*.
